# Malaysian Medical Students’ Career Intention (MMSCI): a cross-sectional study

**DOI:** 10.1186/s12960-024-00939-4

**Published:** 2024-08-22

**Authors:** Zhi Sean Teng, Gerald Tze Zhen Ser, Wei-Han Hong, Chin Hai Teo, Yang Faridah Abdul Aziz, Jamunarani Vadivelu, Malaysia MedEd Collaborative, Jeshua Nathaniel Devan, Nicolas Ong, Shen Hong Law, Shiroshini Periasamy, Sweet Chiao Chean, Subhashini Sivagobi, Yuan Heng Chia, Jack Kang Tan, Yu Xuan Teoh, Eldon Tsai, Jun Jie Oon, Felice Xiao Yuan Yeap

**Affiliations:** 1 Faculty of Medicine, Universiti Malaya, 50603 Kuala Lumpur, Malaysia; 2 Medical Education and Research Development Unit, Faculty of Medicine, Universiti Malaya, 50603 Kuala Lumpur, Malaysia; 3 Department of Primary Care Medicine, Faculty of Medicine, Universiti Malaya, 50603 Kuala Lumpur, Malaysia; 4 Department of Biomedical Imaging, Faculty of Medicine, Universiti Malaya, 50603 Kuala Lumpur, Malaysia; 5 Society of Malaysian Medical Association Medical Students, Malaysian Medical Association, 53000 Kuala Lumpur, Malaysia

**Keywords:** Medical student, Career intention, Career choice, Career perception, Career preference, Emigration

## Abstract

**Background:**

In recent years, there have been many instances of negative sentiments expressed by and resignations observed from doctors working in the Ministry of Health (MOH), Malaysia. However, little is known about the perspectives of medical students and their career intentions. This study aims to determine the current Malaysian medical students’ career intentions immediately after graduation and upon completing the 2 years of housemanship and to establish the factors influencing these intentions.

**Methods:**

This was a cross-sectional study of 859 Malaysian medical students from 21 medical schools who voluntarily completed a self-administered online questionnaire that was disseminated by representatives from medical schools nationwide and social media platforms of a national medical student society.

**Results:**

37.8% of the respondents were optimistic about a career with the Ministry of Health (MOH), Malaysia in the future. Most of the respondents (91.2%) plan to join and complete the MOH Housemanship programme as soon as possible after graduation, with the majority of them (66.2%) planning to complete it in their state of origin. After 2 years of Housemanship programme, only more than half of the respondents (63.1%) plan to continue their careers in MOH. Slightly more than a quarter (27.1%) of the total respondents plan to emigrate to practise medicine, with 80.7% of them planning to return to Malaysia to practise medicine after some years or after completing specialisation training. Combining the career intentions of Malaysian medical students immediately after graduation and upon completion of the 2 years housemanship programme, only a slight majority (57.5%) of the respondents plan to continue their career in MOH eventually. Most of the respondents (85.0%) intend to specialise.

**Conclusion:**

A concerning number of Malaysian medical students plan to leave the Ministry of Health workforce, the main healthcare provider in Malaysia, in the future. Urgent government interventions are needed to address the underlying factors contributing to the potential exodus of future doctors to prevent further straining of the already overburdened healthcare system, posing a significant threat to public well-being. An annual national study to track medical students’ career intentions is recommended to gather crucial data for the human resources for health planning in Malaysia.

**Supplementary Information:**

The online version contains supplementary material available at 10.1186/s12960-024-00939-4.

## Introduction

### Background

Having a career in the medical profession is seen as a prestigious, highly respected and desired profession among students globally [1–3]. Although it is a highly competitive programme to be admitted into, the demand to enter the medical profession is still highly favourable. This is probably due to the ability to secure both a financially and socially rewarding career upon completion of study. There are 32 medical schools in Malaysia [4], both public and private institutions with a vast difference in the fee structure, producing close to 4000 medical graduates annually [5]. Training a medical graduate in a public institution in Malaysia requires a large portion of subsidy by the government to ensure the public is offered a recognised, high-quality yet affordable medical education. Hence, entry requirements into medical school remain to be of high level with an interview put in place in all medical schools to screen the potential students.

Career intentions of medical students may vary according to individual needs, with some wanting to stay for good, pursue a specialty of their choice or delaying specialist training, emigrating to practise medicine abroad, or leaving the profession for other endeavours. As opposed to medical schools abroad which practise graduate entry, medical schools in Malaysia admit undergraduate students who are around 19–20 years old. A medical student typically spends 5 years in medical school after a pre-university programme and then serves 2 years as a house officer (HO) as required under the Medical Act 1971 [6]. As these students mature through medical school, their career intentions may be affected by various factors.

Upon completion of housemanship, the student then obtains his full registration with the Malaysian Medical Council (MMC) [7] and functions as a medical officer (MO). The graduate would then be able to apply for government-sponsored specialist and sub-specialty training thereafter. However, specialist training has been uncertain in recent years due to the contractual appointment of medical officers which does not warrant sponsored specialist training prior to 2022 [8]; while limited seats were offered for contract medical officers from 2022 onwards [9], leaving many graduates considering other options for career advancement [8]. These medical officers will also need to compete with a large pool of applicants consisting of both local and overseas Malaysian doctors for a placement for postgraduate training. The training pathways differ from one specialty to another. Adding to the constraint of the long wait to continue postgraduate training, these medical officers are inundated with a heavy workload and are deemed under-remunerated [8].

The Malaysian public healthcare system has long been grappling with multiple human resource and financial issues, with a report released by the National Audit Department in 2018 which found the Emergency and Trauma Departments in Malaysian public hospitals to be underfunded, understaffed, and overloaded with patients [10]. The medical education landscape in Malaysia has been changing in recent years with the different policies being put in place. There was a delay in housemanship placement, coupled with the appointment to contract post leaving the medical graduates anxious and frustrated. In recent news, the delay in houseman placement has now cascaded to difficulty in recruitment and retaining these housemen. To make matters worse, the maldistribution of doctors to the hospitals has affected training opportunities and manpower in the workforce. Major public hospitals have been reported to have a lack of doctors [8, 11], causing services to be severely affected [12]. While these issues have been highlighted by various societies and movements to support the trainees, there seems to be a delay in providing adequate placement and support for continuing medical education. Furthermore, although the government has recently announced plans to improve these issues, such as raising the on-call allowance rates [13], overhauling human resource policies [14], and establishing a Health Service Commission to reform the public health sector [15, 16], these measures have yet to be implemented.

The career pathways remained uncertain with a lack of urgency from the relevant ministries. This has resulted in many medical graduates looking for other opportunities in other countries which has caused a brain drain to the nation [17]. Although there have been limited studies conducted recently on the career pathway of medical officers in Malaysia, there were even fewer studies focused on medical students’ career intentions, which are crucial for effective policy-making. The current medical students being the major stakeholders in the healthcare fraternity remain the country's asset as they will be the future workforce of the medical fraternity. There is an urgency to study the career intentions of active medical students enrolled in both public and private medical schools in Malaysia to inform the relevant authorities on the direction of these medical graduates which will affect the climate of the Malaysian healthcare workforce in the near future.

### Objectives

This study aims to determine the current medical students’ career intentions immediately after graduation and upon completing the 2 years of housemanship and to establish the motivations behind these intentions.

## Methods

### Study design and setting

The Malaysian Medical Student Career Intention (MMSCI) study was a national and multi-centre cross-sectional online survey. This study involved participants from medical schools in Malaysia recognised by the Malaysian Medical Council (MMC) under the Second Schedule Medical Act 1971 [4] using a non-probable (convenience) sampling method. The data collection was conducted from the 5th of May 2023 to the 15th of October 2023.

### Participant eligibility and recruitment

All medical students of Malaysian nationality who are registered in a public or private medical school recognised by the MMC and operating in Malaysia were eligible to join this study. The students must also complete the entire medical programme locally in Malaysia. Those completing pre-clinical years overseas but subsequently clinical years in Malaysia were also included.

To achieve a representative sample and improve the generalisation of the findings, a collaborative network of medical students—the ‘Malaysia MedEd Collaborative’ was established in collaboration with the Society of Malaysian Medical Association Medical Students (SMMAMS). An attempt to recruit collaborators from all universities was made through an open recruitment process via SMMAMS. A total of 30 collaborators were recruited from 18 out of 32 medical schools, with a maximum of 2 collaborators per medical school (1 pre-clinical year medical student and 1 clinical year medical student), before the study launch. All recruited collaborators were then given an online briefing session regarding the study’s aims and details, including the specifics of the questionnaire itself. The collaborators were then provided with the link to the online questionnaire hosted on Google Forms and were responsible for distributing the survey within their respective medical schools using various distribution channels such as social media, internal email communication, and word of mouth. Additionally, an invitation to participate was also distributed by SMMAMS through their various communication channels such as social media, internal newsletter, and internal email communication to reach the wider Malaysian medical students population. The sample size calculation using OpenEpi software determined that at least 377 responses were required to achieve a representative sample with a confidence level of 95% and 0.05 margin of error, based on an estimated student population of 20,000 (4000 medical students per year) in Malaysia. We multiplied the sample size by the predicted design effect of two to account for the use of convenience sampling and online survey [18]. Hence, the minimum survey sample size was 754 (377 × 2). However, in this study, we aimed to recruit at least 50 participants per medical school to ensure fair representativeness across medical schools.

### Study instrument and data collection

A self-administered, 51-item online questionnaire was developed based on literature and hosted on Google Forms. The content of the questionnaire was validated by experts from the field of medical education. The questionnaire was designed in English language, as English language is used as the medium of instruction in all medical schools in Malaysia. The questions were formulated using Likert scale matrices, multiple-choice selections, and open-text entries. We then pilot-tested the questionnaire with nine medical students to assess its feasibility and relevance of the questionnaire. Feedback from the pilot study was collected to improve the questionnaire.

The questionnaire consists of four sections: (A) Demographic information; (B) Career outlook in the Ministry of Health (MOH), Malaysia; (C) Career Intentions after graduation and housemanship; (D) Specialisation Intentions. The complete questionnaire is provided in Supplementary File 1: MMSCI Questionnaire.

Each participant was informed of the purpose of the research and provided informed consent. All data from this study were stored in a secure password-protected cloud-based storage service account specifically created for this study and is only accessible by the core team.

### Data analysis

A total of 882 responses from 21 medical schools were collected. Incomplete entries with missing data were excluded. Duplicated entries with the same name, IC or email were eliminated before analysis, with the earliest entry retained. Entries without informed consent and from international medical students were removed. Finally, 859 responses from 21 medical schools were included for analysis.

The IBM SPSS Statistics for Windows, version 29 (IBM Corp., Armonk, N.Y., USA) was used to run the statistical analysis. Descriptive statistics mainly proportion was used to present the outcomes. Age was reported using median and interquartile range as it was not normally distributed. For items with a 5-point Likert scale, the lowest 2 points (e.g. very unsatisfied and unsatisfied) and the highest 2 points (e.g. satisfied and very satisfied) were combined and presented with the neutral score.

## Results

### Demographics

A total of 859 medical students in Malaysia responded to this survey and their demography can be found in Table 1. Most of the respondents are female (64.4%), Chinese (41.3%) and from the state of Selangor (25.8%). The respondents are quite fairly distributed in terms of type of university: public (54.4%) vs private (45.6%); and year of study: pre-clinical (46.1%) vs clinical (53.9%). The median age of respondents was 22 (interquartile range = 2).

**Table 1:** Demographic profile of participants

Characteristics	*n* (%)
*Gender*	
Male	298 (34.7)
Female	553 (64.4)
Non-binary	3 (0.3)
Prefer not to say	5 (0.6)
*Ethnicity*	
Malay	268 (31.2)
Chinese	355 (41.3)
Indian	187 (21.8)
Others	49 (5.7)
*State of origin*	
Selangor	222 (25.8)
Penang	101 (11.8)
Kuala Lumpur	94 (10.9)
Perak	84 (9.8)
Sarawak	72 (8.4)
Johor	68 (7.9)
Kedah	51 (6.9)
Kelantan	38 (4.4)
Malacca	31 (3.6)
Sabah	27 (3.1)
Negeri Sembilan	25 (2.9)
Pahang	23 (2.7)
Terengganu	19 (2.2)
Perlis	2 (0.2)
Putrajaya	2 (0.2)
*Type of university*	
Public	467 (54.4)
Private	392 (45.6)
*Year of study*	
Year 1	201 (23.4)
Year 2	195 (22.7)
Year 3	158 (18.4)
Year 4	233 (27.1)
Year 5	72 (8.4)
*Year of study*	
Pre-clinical	396 (46.1)
Clinical	463 (53.9)
*Age*	
Median (interquartile range)	22 (2)

### Career perception and intention upon graduation

In terms of career perception upon graduation, 37.8% of the respondents were optimistic about a career with MOH in the future. Of all aspects of a career with MOH, satisfaction ranges from 8.0 to 37.4%. The most satisfied aspects are clinical exposure and general clinical skills training opportunities (37.4%), quality of specialisation training (33.3%), and duration of specialisation training (29.7%). There were six aspects which more than half of the respondents reported being unsatisfied with, which include work–life balance (67.6%), working hours (65.8%), job security (57.5%), working culture (57.0%), working environment and conditions (57.0%), and remuneration and pay at the houseman (HO) and medical officer level (51.1%) (Table 2).

**Table 2:** Career perception and intention upon graduation

	Question	*n* (%)		
1	*What do you think about your future in general as a doctor in MOH? (N = 859)*	*Optimistic*	*Neutral*	*Pessimistic*
		325 (37.8)	337 (39.2)	197 (22.9)
2	*Views on a career with MOH?* (*N* = 859)	*Satisfied*	*Neutral*	*Unsatisfied*
	Clinical exposure and general clinical skills training opportunities	321 (37.4)	341 (39.7)	197 (22.9)
	Quality of specialisation training	286 (33.3)	434 (50.5)	139 (16.2)
	Duration of specialisation training	255 (29.7)	421 (49.0)	183 (21.3)
	Remuneration and pay at the specialist level	222 (25.8)	405 (47.1)	232 (27.0)
	Duration of service before entering a specialisation training programme	206 (24.0)	393 (45.8)	260 (30.3)
	Overall satisfaction with the prospect of working with MOH	163 (19.0)	368 (42.8)	328 (38.2)
	Career pathway clarity	170 (19.8)	303 (35.3)	386 (44.9)
	Opportunities to enter a specialisation training programme (competition)	160 (18.6)	296 (34.5)	403 (46.9)
	Job security	126 (14.7)	239 (27.8)	494 (57.5)
	Ability to choose a work location/department	119 (13.9)	327 (38.1)	413 (48.1)
	Working culture	112 (13.0)	257 (29.9)	490 (57.0)
	Working environment and conditions	111 (12.9)	258 (30.0)	490 (57.0)
	Remuneration and pay at the Houseman (HO), Medical Officer (MO) level	106 (12.3)	314 (36.6)	439 (51.1)
	Work–life balance	77 (9.0)	201 (23.4)	581 (67.6)
	Working hours	69 (8.0)	225 (26.2)	565 (65.8)
3	*Are you planning to join and complete the MOH Housemanship programme as soon as possible after graduation?*	*N= 859*		
	Yes	783 (91.2)		
	No	76 (8.9)		
	Emigrate to practise overseas	58 (6.8)		
	Take a break from clinical practice temporarily (no more than 3 years)	17 (2.0)		
	Leave clinical practice permanently	1 (0.1)		
4	*Which state do you plan to pursue your housemanship?*	*N = 783*		
	Kuala Lumpur	166 (21.2)		
	Selangor	157 (20.1)		
	Sabah	74 (9.5)		
	Sarawak	74 (9.5)		
	Penang	62 (7.9)		
	Johor	54 (6.9)		
	Perak	53 (6.8)		
	Kelantan	31 (4.0)		
	Kedah	30 (3.8)		
	Negeri Sembilan	23 (2.9)		
	Malacca	18 (2.3)		
	Pahang	17 (2.2)		
	Terengganu	16 (2.0)		
	Putrajaya	4 (0.5)		
	Perlis	2 (0.3)		
	Labuan	2 (0.3)		
5	*Is the above choice your state of origin/ hometown?*	*N = 783*		
	All participants	*N* = 783		
	Yes	518 (66.2)		
	No	265 (33.8)		
	Excluding participants from KL & Selangor	*N* = 499		
	Yes	322 (64.5)		
	No	177 (35.5)		
6	*Do you understand how the Housemanship (HO) programme works and know what is expected from you in housemanship?*	*N = 859*		
	Yes	407 (47.4)		
	No	61 (7.1)		
	Maybe	391 (45.5)		
7	*Do you think that your medical school has/ will train and prepare you adequately for housemanship?*	*N = 859*		
	Yes	608 (70.8)		
	No	251 (29.2)		
8	*Specialisation intention*	*N = 859*		
	Yes	730 (85.0)		
	No	5 (0.6)		
	Maybe	124 (14.4)		

Most of the respondents were planning to join and complete the MOH Housemanship programme as soon as possible after graduation (91.2%), with the majority choosing to pursue their housemanship in Kuala Lumpur (21.2%), followed by Selangor (20.1%), Sabah (9.5%) and Sarawak (9.5%). The majority of the respondents chose to complete their housemanship in their state of origin (66.2%). Of our responses, almost half (47.4%) understand how the Housemanship programme works and know what is expected from them in housemanship, and a majority of them think that they are adequately prepared for housemanship by their medical school (70.8%). Most of our respondents intend to specialise (85.0%) (Table 2).

### Medical students’ career intention after housemanship

After completing the MOH Housemanship programme, slightly more than half of our respondents (63.1%) chose to continue their careers in MOH. The most important reasons for those who chose to remain in the MOH include clinical exposure and general clinical skills training opportunities (84.8%), opportunities to enter a specialisation training programme (83%), and job security (82.4%). The least important reasons were remuneration and pay at the Houseman (HO) and Medical Officer (MO) level (67.2%), work–life balance (69.4%), and remuneration and pay at the specialist level (71.5%). For those who chose not to continue their career in MOH after completing housemanship, the top reason was to emigrate and practise overseas (22.3%) (Table 3).

**Table 3:** Medical students’ career intention after housemanship

	Question	*n* (%)		
1	*I plan to complete my MOH Housemanship programme and *(*N* = 783)			
	Continue my career in MOH	494 (63.1)		
	Emigrate to practise overseas	175 (22.3)		
	Move out to private practice	110 (14.0)		
	Leave clinical practice permanently	4 (0.5)		
2	*Reasons for continuing career with MOH (N = 494)*	*Important*	*Neutral*	*Not Important*
	Clinical exposure and general clinical skills training opportunities	419 (84.8)	68 (13.8)	7 (1.4)
	Opportunities to enter a specialisation training programme (competition)	410 (83.0)	76 (15.4)	8 (1.6)
	Job security	407 (82.4)	76 (15.4)	11 (2.2)
	Career pathway clarity	403 (81.6)	81 (16.4)	10 (2.0)
	Quality of specialisation training	400 (81.0)	85 (17.2)	9 (1.8)
	Duration of service before entering a specialisation training programme	386 (78.1)	98 (19.8)	10 (2.0)
	Ability to choose a work location as MO	375 (75.9)	100 (20.2)	19 (3.8)
	Working environment and conditions	373 (75.5)	100 (20.2)	21 (4.3)
	Duration of specialisation training	371 (75.1)	111 (22.5)	12 (2.4)
	Personal reasons (family, partner, spouse, etc.)	371 (75.1)	106 (21.5)	17 (3.4)
	Working culture	364 (73.7)	106 (21.5)	24 (4.9)
	Remuneration and pay at the specialist level	353 (71.5)	121 (24.5)	20 (4.0)
	Work–life balance	343 (69.4)	118 (23.9)	33 (6.7)
	Remuneration and pay at the Houseman (HO), Medical Officer (MO) level	332 (67.2)	136 (27.5)	26 (5.3)

### Medical students’ emigration intention to pursue medical career

Of all the 859 respondents in this survey, slightly more than a quarter plan to emigrate (27.1%). Among those who planned to emigrate, about a quarter (24.9%) planned to immediately emigrate after graduation from medical school while 75.1% planned to emigrate after completing housemanship. In terms of country, most chose Australia (27.5%), the United Kingdom (25.7%) and Singapore (14.4%) as the destination emigration countries. The most important reasons for emigration include working environment and conditions (92.7%), job security (92.3%), and working culture (91.4%). The least important reasons were personal reasons (68.2%), ability to choose a work location (76.4%), and duration of specialisation training (85.4%) (Table 4).

**Table 4:** Medical students’ emigration intention to pursue medical career

Question	*n* (%)		
*Emigration statistics*	*N= 859*		
Do not plan to emigrate	626 (72.9)		
Planning to emigrate	233 (27.1)		
Stage of career	*N* = 233		
Immediately after Graduation	58 (24.9)		
After completion of housemanship	175 (75.1)		
Intended emigration countries*			
Australia	78		
United Kingdom	73		
Singapore	41		
United States of America	29		
New Zealand	13		
Ireland	10		
Canada	6		
Other EU countries	3		
Spain	2		
United Arab Emirates	2		
China	2		
South Korea	2		
Germany	1		
Netherlands	1		
Norway	1		
Switzerland	1		
Finland	1		
Taiwan	1		
Japan	1		
India	1		
Brunei	1		
Saudi Arabia	1		
Pakistan	1		
Other Middle East	1		
Undecided	11		
*Plans to return to Malaysia after emigration*	*N = 233*		
Yes, after completion of specialisation training	93 (39.9)		
Yes, after some years	95 (40.8)		
No	45 (19.3)		
*Reasons for emigrating to practise overseas (N = 233)*	*Important*	*Neutral*	*Not Important*
Working environment and conditions	216 (92.7)	14 (6.0)	3 (1.3)
Job security	215 (92.3)	15 (6.4)	3 (1.3)
Working culture	213 (91.4)	19 (8.2)	1 (0.4)
Quality of specialisation training	212 (91.0)	20 (8.6)	1 (0.4)
Living environment or standard of living	210 (90.1)	21 (9.0)	2 (0.9)
Remuneration and pay at the specialist/ consultant level	209 (89.7)	21 (9.0)	3 (1.3)
Career pathway clarity	209 (89.7)	21 (9.0)	3 (1.3)
Opportunities to enter a specialisation training programme (competition)	209 (89.7)	20 (8.6)	4 (1.7)
Remuneration and pay at junior level	208 (89.3)	24 (10.3)	1 (0.4)
Work–life balance	208 (89.3)	22 (9.4)	3 (1.3)
Clinical exposure and general clinical skills training opportunities	206 (88.4)	26 (11.2)	1 (0.4)
Duration of service before entering a specialisation training programme	200 (85.8)	30 (12.9)	3 (1.3)
Duration of specialisation training	199 (85.4)	28 (12.0)	6 (2.6)
Ability to choose a work location	178 (76.4)	48 (20.6)	7 (3.0)
Personal reasons (family, partner, spouse, etc.)	159 (68.2)	60 (25.8)	14 (6.0)

*Respondents may provide more than 1 answer

The majority (80.7%) of them planned to return to Malaysia after some years or after completing specialisation training (Table 4).

### Medical students’ career outside of MOH, Malaysia

Of the 783 respondents who plan to complete their housemanship programme in Malaysia, 110 (14.0%) plan to move to private practice upon completion of housemanship (Table 3). Among the reasons for moving to private practice, the top three most important reasons included working environment and conditions (96.4%), ability to choose a work location (95.4%), and remuneration and pay (93.6%) (Table 5).

**Table 5:** Medical students’ career outside of MOH, Malaysia

	Question	*n* (%)		
1	*Career outside of MOH, Malaysia*	*N= 859*		
	Plan to stay in MOH	494 (57.5)		
	Take a break from clinical practice temporarily after graduation (less than 3 years)	17 (2.0)		
	Career outside of MOH	348 (40.5)		
	*Plans of a career outside MOH*	*N= 348*		
	Emigration (upon graduation and after housemanship)	233 (67.0)		
	Private practice (after housemanship)	110 (31.6)		
	Leaving clinical medicine permanently (upon graduation and after housemanship)	5 (1.44)		
2	*Private practice (after Housemanship) statistics*	*N = 110*		
	*Reasons for moving to private practice (N = 110)*	*Important*	*Neutral*	*Not Important*
	Working environment and conditions	106 (96.4)	4 (3.6)	0 (0.0)
	Ability to choose a work location	105 (95.4)	5 (4.5)	0 (0.0)
	Remuneration and pay	103 (93.6)	6 (5.5)	1 (0.9)
	Work–life balance	103 (93.6)	6 (5.5)	1 (0.9)
	Working culture	102 (92.7)	8 (7.3)	0 (0.0)
	Job security	96 (87.3)	14 (12.7)	0 (0.0)
	Personal reasons (family, partner, spouse, etc.)	96 (87.3)	11 (10.0)	3 (2.7)
	Career pathway clarity	96 (87.3)	11 (10.0)	3 (2.7)

Combining the career intentions of Malaysian medical students immediately after graduation and upon completion of the 2 years housemanship programme, only a slight majority (57.5%) of our respondents plan to continue their career in MOH eventually. Of those who chose a career outside of MOH, two-thirds chose to emigrate (67.0%) (Table 5).

## Discussion

### Perception of working in MOH

In recent years, a large number of Malaysian doctors (including specialists) have resigned from public service [19], while resignations among contract medical officers have risen by 1131% in 2022 compared to 2017 [20]. A total of 1696 medical officers have resigned from their positions in MOH in 2022 alone [21].

According to a recent poll conducted by CodeBlue in early 2023 [22], the majority of Malaysian government healthcare workers (inclusive of doctors, pharmacists, dentists, nurses, assistant medical officers, and allied healthcare workers) have been shown to be unsatisfied with the situation of the public healthcare system (98%), with almost three-quarters of them thinking of quitting the public healthcare sector (73%). A majority of government healthcare workers also noted that they felt they were underpaid (80%), overworked (78%), and insecure about their career progression (61%). The findings in our study were consistent with the general sentiment among public healthcare workers, where only a minority of our study participants were optimistic about a career with MOH (37.8%), and a majority were unsatisfied with the remuneration and pay at the HO and MO level (51.1%), working hours (65.8%), and job security (57.5%). This suggests that the sentiment towards Malaysian public healthcare was similar between both doctors and medical students.

### Medical brain drain

Physician migration, often termed “brain drain”, is a global phenomenon, particularly noticeable from LMIC to HIC. A study from the UK suggested that 32.25% of medical students intended to emigrate to practise medicine abroad [23]; 33% of Pakistan medical students plan to leave Pakistan after graduation [24]; 45% of Indian medical students planned to pursue their residency abroad [25]; 49% of Ghanaian medical students had intentions to migrate after school [26]; 70.1% of Moroccan medical students had intention to leave the country [27]; 88% of Irish medical students indicated they were either definitely migrating or contemplating migrating following graduation or completion of the pre-registration intern year [28]. In comparison, Malaysian medical students, situated in a UMIC, show a moderate prevalence in their intention to emigrate. Globally, the most popular destinations for physician migration are the USA and the UK [29]. Our findings reveal that the migration destination intentions of Malaysian medical students align with global trends. Australia received the highest preference in our study, mirroring a trend seen in the UK and Ireland [23, 28].

It is shown that the medical brain drain is a complicated phenomenon resulting from push, pull and dyadic factors [29]. According to our study, over a quarter of the respondents intend to emigrate. Their decision to emigrate and pursue the early stages of their career in other countries is largely influenced by the working environment, job security, and working culture that MOH can provide. However, a significant majority (80.7%) of them plan to return to Malaysia to practise later on, with almost 40% of them planning to do so after completion of specialisation. A potential conclusion drawn from these results would be that Malaysian medical students would still desire to practise medicine in Malaysia; push factors play a huge role in their decision. Despite the majority intending to return to Malaysia after training abroad, their homecoming plans may face changes over the years due to various factors. More studies are required on this aspect.

### Projected doctors’ workforce in MOH, Malaysia

In 2022, the number of medical graduates fell by 57% to 4875 from 8511 in 2019, attributed to the deferral of studies by medical students who did not complete their studies in the expected timeframe [30]. One of the reasons could be due to the interruption of studies by the COVID-19 pandemic. Currently, it is estimated that Malaysian local medical schools will produce about 4000 medical graduates yearly with the admission quota of 4820 yearly imposed by the MOH [5]. Additionally, 1000 to 2000 overseas medical graduates return to Malaysia yearly, [31] summing up to around 6000 Malaysian medical graduates. Considering our study findings of 91.2% of our respondents’ intention of commencing their housemanship as soon as possible upon graduation, we expect a yearly intake of approximately 3648 local graduates, and 2000 overseas medical graduates, totalling around 5600 HO intake per year. This suggests a potential return to the pre-pandemic HO appointments level of 5000 to 6000 per year, addressing the current healthcare services limitation [30]. However, based on the low number of HO intake of 3271 in 2023, which has yet to show signs of an increase of intake from 2022, and compared to our estimated yearly HO intake of 5600, one should wonder whether the persistently low intake number in the recent years is solely due to the interruption of studies as reported. Hence, this again highlights the importance of an annual national medical graduate employment survey to closely follow the career intentions of Malaysian medical students.

Furthermore, there is also a maldistribution of doctors in Malaysia, especially between the rural and urban areas, with noticeable shortages in East Malaysia (Sabah and Sarawak). This imbalance is partly due to the reluctance of medical workers to practise in these areas of Malaysia [32]. Our study shows that the majority of respondents would like to complete their housemanship training in their state of origin. “Being close to home” has previously been listed as the top criterion for Malaysian medical graduates when selecting a hospital for their housemanship training [33]. Similar findings were also reported with medical students in other countries such as Germany [34], and Nepal [35]. Strong evidence has also shown that medical students with a rural origin were more likely to return to rural areas to practise medicine [36–38]. Given this evidence, we suggest policymakers explore strategies to increase the intake of medical students from rural origins, especially in Sabah and Sarawak and retain them in rural Malaysia. Interesting Japanese policies such as “Jichi” and “Chiikiwaku” have been proven effective in increasing the number of physicians in rural settings by providing education scholarships to medical students of rural origin that cover their medical education tuition. In return, Jichi graduates are expected to work in their home prefectures for 9 years to get their undergraduate tuition fee waived [39]. Other successful approaches include long rural immersion during medical school [40].

Upon completion of housemanship, a houseman will then progress to becoming a medical officer (MO), constituting the largest group of doctors within the MOH [41]. Our study reports that only 63.1% of medical students intend to stay within the Ministry of Health upon completion of housemanship, reflecting the current trend of more than 54% of MOs resigning in 2022 [21]. This is indeed alarming as public healthcare remains the main healthcare provider for most Malaysians, with 50.1% of the Malaysian population not having any means of supplementary financial coverage for medical treatment apart from tax-funded healthcare services by the government as highlighted by the National Health and Morbidity Survey (NHMS) 2019 [42]. With an increased trend in the prevalence of NCDs [42] and as Malaysia progresses to become an aged nation by 2044 [43], the nation’s demand for public healthcare services will undoubtedly increase. Hence, the government should take immediate action to address the dissatisfaction of doctors and the healthcare workforce in general to mitigate and reverse the alarming trend of doctors leaving MOH [44]. Focused attention is suggested on aspects that more than half of our respondents reported being unsatisfied with such as work–life balance, working hours, job security, working culture, working environment and conditions, and remuneration and pay at the HO and MO levels. Recognising the complexity of this issue and its multi-stakeholder nature, the call to action is urgent. The urgency arises from the potential risk of further straining the already overburdened healthcare system, posing a significant threat to public well-being.

Lastly, it is known that Malaysia requires more medical specialists to support an increasing demand for health services. As of September 2021, there are 13,000 medical specialists across both public and private healthcare, yet the projection of needing 28,000 specialists by 2030 seems unattainable [45, 46]. Our study revealed that a high 85% of medical students intend to specialise. Hence, efforts should be taken to retain and train these aspiring medicos. A comprehensive analysis of the specialisation perception and intention of Malaysian medical students from this study will be published in the future.

### Study limitation

There are several limitations in this study. Firstly, there was no representation from a small number of medical schools, potentially limiting the generalisation of our study. Selection bias may be present as the study may attract more students interested in this topic or better informed about Malaysian healthcare affairs. Some students may not have received invitations to this study or been made aware of this study, and others may have chosen not to participate. Furthermore, we intentionally excluded the option of pursuing a future career with the Ministry of Higher Education (MOHE) in university hospitals and the Ministry of Defence (MOD) in military hospitals while designing the study. This may lead to inaccuracy of the study as some may be interested in serving in MOHE or MOD. However, it is worth noting that the number of doctors working in MOHE and MOD is relatively small compared to those working with the MOH. Finally, it should be stressed that our respondents were medical students, and their current perceptions, as reported, might be influenced by various factors such as their limited knowledge of the realities of working as a doctor. These perceptions could evolve as they progress in their career and life stages.

### Importance and implication

Some nations, such as Australia, New Zealand, [47] the United Kingdom [23, 48], the United Arab Emirates [49], and Malawi [50], possess robust literatures that have generated significant research outputs related to medical students’ career intentions. These literatures may have directly or indirectly shaped their medical education and health workforce policies [51]. The current limited literatures on this topic in Malaysia are usually single-centre [52, 53], which restrict the diversity and generalisation of the findings; have small sample sizes, reducing the statistical power and reliability of the findings; are dated [54] and lack comprehensiveness on career intentions [55, 56]. This study stands as the largest and most comprehensive investigation into Malaysian medical students’ career intentions, involving the majority of the medical schools in Malaysia. Hence, the findings of this study will address some of these knowledge gaps and provide comprehensive insights into Malaysian medical students’ career intentions. It is hoped that the findings will provide evidence-based value to the leaders and policymakers of MOH in the planning of human resources for health (HRH) and the ongoing health reforms.

## Conclusion

In conclusion, the general perception and sentiment of Malaysian medical students regarding a future career with the MOH, Malaysia is varied. Most medical students would like to complete their housemanship programme in Malaysia, with the majority preferring to do so in their state of origin/ hometown. More than a quarter of medical students would like to emigrate overseas to practise, mostly upon completion of housemanship. Alarmingly, over 40% of Malaysian medical students would prefer a career outside of MOH, with most planning to leave MOH upon completion of housemanship. This study underscores the need for urgent government intervention to address the underlying factors contributing to the exodus of doctors, ultimately mitigating this alarming trend.

In light of the study’s insights, an annual, national, and large-scale survey on Malaysian medical students’ career intentions is recommended. Focused qualitative studies could further explore, explain, and validate the findings of this study. This is to ensure continual engagement with the future employees of MOH, providing invaluable data for the ongoing healthcare workforce planning and contributing to the long-term sustainability of Malaysia’s healthcare system.

### Supplementary Information

Supplementary Material 1.

## Data Availability

The datasets used and/or analysed during the current study are available from the corresponding author upon reasonable request.

## Abbreviations

EU: European Union
HIC: High income country
HO: House officer
HRH: Human Resources for Health
IC: Identity card
KL: Kuala Lumpur
LMIC: Low and middle income country
MMC: Malaysian Medical Council
MMSCI: Malaysian Medical Students’ Career Intention
MO: Medical officer
MOD: Ministry of Defence
MOH: Ministry of Health
MOHE: Ministry of Higher Education
NCD: Non-communicable disease
NHMS: The National Health and Morbidity Survey
SMMAMS: Society of Malaysian Medical Association Medical Students
SPSS: Statistical Package for Social Sciences
UK: United Kingdom
UMIC: Upper middle-income country
USA: United States of America
