# Supplementary material for: Malaysian Medical Students’ Career Intention (MMSCI): a cross-sectional study
Source: Hum Resour Health. 2024 Aug 22;22:59. doi: 10.1186/s12960-024-00939-4 (PMC11340133; doi:10.1186/s12960-024-00939-4)
Supplement: Supplementary file 1 — Supplementary Material 1. [file 12960_2024_939_MOESM1_ESM.pdf]

UNIVERSITI MALAYA

MALAYSIAN MEDICAL ASSOCIATION

SOCIETY OF MALAYSIAN MEDICAL ASSOCIATION MEDICAL STUDENTS

Malaysian Medical Student Career Intentions: A Cross-Sectional Study

Questionnaire

The questionnaire consists of four sections: A) Demographic information; B) Career outlook in the Ministry of Health (MOH), Malaysia; C) Career Intentions after graduation and housemanship; D) Specialisation Intentions.

In Section A, the demographic information includes the University Student ID number; Age; Date of Birth; Gender; National Identity Card Number (IC); Ethnicity; State of Origin; University; Year of Study; and Expected Year of Graduation. Section B explored the general views and perceptions of medical students working for the MOH as doctors, and their views on how their medical schools have or will prepare them to be doctors in the future. In Section C, participants are asked to indicate their intended career pathways immediately after graduation and after their housemanship programme (if applicable). Section D surveyed the current specialty intentions of medical students. Participants were also inquired about the level of commitment to their decisions and the factors that played a role in shaping those decisions for both Section C and D. All participants were to complete both Section A and Section B. The subsequent questions relied on the responses to previous questions.

Instructions:

- This questionnaire consists of 4 sections:
    - Section A: Demographics
    - Section B: Career Outlook in MOH
    - Section C: Career Intentions
    - Section D: Specialisation
- Please answer all the compulsory questions
- Please select your answer(s) and fill up the blank

Section A: Demographics

Student Matrix Number
This information will be solely used to verify your student status only and will only be accessible by the core research team.

Age

Date of Birth

Gender
- Male
- Female
- Non-binary
- Prefer Not to say
- Others

Ethnic
- Malay
- Chinese
- Indians
- Others

State of Origin
- Johor
- Kedah
- Kelantan
- Kuala Lumpur
- Labuan
- Malacca
- Negeri Sembilan
- Pahang
- Penang
- Perak
- Perlis
- Putrajaya
- Sabah
- Sarawak
- Selangor
- Terengganu

University
1. Asian Institute of Medicine, Science and Technology University (AIMST)
2. Universiti Islam Antarabangsa Sultan Abdul Halim Mu'adzam Shah (UNISHAMS)
3. Universiti Kuala Lumpur Royal College of Medicine Perak (UniKL-RCMP)
4. Quest International University Perak (QIUP)
5. Royal College of Surgeons in Ireland & University College Dublin (RUMC/PMC)
6. Universiti Malaya (UM)
7. Universiti Kebangsaan Malaysia (UKM)
8. International Medical University (IMU)
9. Monash University Malaysia (Monash)
10. Universiti Pertahanan National Malaysia (UPNM)

11. Mahsa University (MAHSA)
12. Perdana University (PERDANA)
13. Universiti Putra Malaysia (UPM)
14. Universiti Teknologi Mara (UiTM)
15. University of Cyberjaya (UoC/CUCMS)
16. Management and Science University (MSU)
17. SEGi University & Colleges (SEGi)
18. Universiti Tunku Abdul Rahman (UTAR)
19. Taylor's University (Taylor's)
20. Lincoln University (LINCOLN)
21. Universiti Sains Malaysia (USM)
22. Universiti Islam Antarabangsa Malaysia (IIUM)
23. Widad University College (WIDAD)
24. Universiti Sultan Zainal Abidin (UniSZA)
25. Newcastle University Medicine Malaysia (NUMed)
26. Asia Metropolitan University (AMU)
27. Melaka Manipal Medical College (MMMC/MUCM)
28. Universiti Sains Islam Malaysia (USIM)
29. UCSI University (UCSI)
30. Universiti Malaysia Sabah (UMS)
31. Universiti Malaysia Sarawak (UNIMAS)

Year of Study
Please indicate your year of study in the academic year 2022/2023.

- Year 1
- Year 2
- Year 3
- Year 4
- Year 5
- Year 6

Expected Year of Completion of Study

- 2023
- 2024
- 2025
- 2026
- 2027
- 2028

Section B: Career Outlook in MOH

What do you think about your future in general as a doctor in MOH?

- Very optimistic
- Optimistic
- Neutral
- Pessimistic
- Very pessimistic

Do you understand how the HO programme works and know what is expected from you in housemanship?

- Yes
- Maybe
- No

Do you think that your medical school has/ will train and prepare you adequately for housemanship?

- Yes
- No

Which aspect do you think that medical school could improve on in preparing you for housemanship?
(Rating from a scale of 1 to 4 for each aspect)

- Clinical Exposure
- Clinical Skills Training
- Basic Medical Science Knowledge
- Research
- Communication skills
- Leadership skills
- Professionalism
- Skills needed to perform daily tasks in housemanship (e.g. referral call)
- Building Resilience

If given a second chance, would you consider doing medicine again?

- Yes
- No

If no, which course would you pursue instead?

Views on a career with MOH?
For each of the aspects below, please rate your perceived satisfaction towards the current status of a career in MOH.
(very satisfied, satisfied, neutral, unsatisfied, not satisfied at all)

- Remuneration and pay at the Houseman (HO), Medical Officer (MO) Level
- Remuneration and pay at the Specialist Level
- Job Security
- Working environment and conditions
- Working culture
- Work-life balance
- Working hours
- Ability to choose a work location/department

- Career pathway clarity
- Clinical exposure and general clinical skills training opportunities
- Opportunities to enter a specialisation training programme (competition)
- Duration of specialisation training
- Quality of specialisation training
- Overall satisfaction with the prospect of working with MOH
- Duration of service before entering a specialisation training programme

Any suggestions/ opinions on how to improve the prospects of working in MOH?
(optional)

Section C: Career Intentions

Are you planning to join and complete the MOH Housemanship programme ASAP after graduation?

- Yes
- No

If yes, I plan to complete my MOH Housemanship Programme and

- Continue my career in MOH
- Emigrate to practice overseas
- Move out to private practice
- Leave clinical practice permanently

If not, I plan to

- Emigrate to practice overseas
- Leave clinical practice permanently
- Take a break from clinical practice temporarily (no more than 3 years)

<u>To join and complete MOH Housemanship programme</u>

Reasons to join and complete Housemanship Programme
Please indicate the level of importance of these factors in contributing to your decision (Very important, Important, Neutral, Not Important, Not important at all)

- Remuneration and pay at Houseman (HO) and Medical Officer (MO) level
- Job Security
- To gain full registration with Malaysian Medical Council
- Working environment and conditions
- Working culture
- Work-life balance
- Ability to choose a work location for HO
- Personal reasons (Family, partner, spouse, etc)
- Career pathway clarity
- Clinical exposure and general clinical skills training opportunities
- Career Progression Opportunities

Which state do you plan to pursue your housemanship?
- Johor
- Kedah
- Kelantan
- Kuala Lumpur
- Labuan
- Malacca

- Negeri Sembilan
- Pahang
- Penang
- Perak
- Perlis
- Putrajaya
- Sabah
- Sarawak
- Selangor
- Terengganu

Is the above choice your state of origin/ hometown?

- Yes
- No

<u>**Continuing Career with MOH**</u>

You have indicated that you would like to continue your career with MOH. How serious are you regarding this decision?

- It is just a thought in my mind
- I am actively searching for important and relevant info
- I am in the process of detailed planning
- I have made concrete steps or actions that will pave the way for this decision after the completion of my HO

Reasons for continuing your career with MOH

Please indicate the level of importance of these factors in contributing to your decision (Very important, Important, Neutral, Not Important, Not important at all)

- Remuneration and pay at the HO MO level
- Remuneration and pay at the specialist level
- Job Security
- Working environment and conditions
- Working culture
- Work-life balance
- Ability to choose a work location for MO
- Personal reasons (Family, partner, spouse, etc)
- Career pathway clarity
- Clinical exposure and general clinical skills training opportunities
- Opportunities to enter a specialisation training programme (competition)
- Duration of specialisation training
- Quality of specialisation training
- Duration of service before entering a specialisation training programme

Do you plan to specialise?

- Yes
- No
- Maybe

<u>Emigrating to Overseas</u>
You have indicated that you would like to emigrate to practice overseas. Which country are you planning to emigrate to?

You have indicated that you would like to emigrate to practice overseas. How serious are you regarding this decision?

- It is just a thought in my mind
- I am actively searching for essential and relevant info
- I am currently applying/ preparing/ have set for overseas medical licensing exams (e.g. PLAB, USMLE, AMC exams, etc)
- I do not need any overseas licensing exams, and I am or will be applying for/getting a job offer from overseas.

Is your medical school recognised by other Medical councils outside of Malaysia?

- Yes
- No
- I am not sure

Reasons for emigrating to practice overseas
Please indicate the level of importance of these factors in contributing to your decision (Very important, Important, Neutral, Not Important, Not important at all)

- Remuneration and pay at the junior level
- Remuneration and pay at the specialist/ consultant level
- Job Security
- Working environment and conditions
- Working culture
- Work-life balance
- Living environment or standard of living
- Ability to choose a work location
- Personal reasons (Family, partner, spouse, etc)
- Career pathway clarity
- Clinical exposure and general clinical skills training opportunities
- Opportunities to enter a specialisation training programme (competition)
- Duration of specialisation training
- Quality of specialisation training
- Duration of service before entering a specialisation training programme

Do you plan to return to Malaysia to practice?

- Yes, after completion of my specialisation training
- Yes, after some years
- No

<u>Moving to Private Practice</u>
You have indicated that you would like to move to private practice. What do you plan to do?

You have indicated that you would like to emigrate and move to private practice. How serious are you regarding this decision?

- It is just a thought in my mind
- I am actively searching for important and relevant info
- I am in the process of detailed planning
- I have made concrete steps or actions that will pave the way for this decision in the future after the completion of my HO

Reasons for moving to private practice
Please indicate the level of importance of these factors in contributing to your decision (Very important, Important, Neutral, Not Important, Not important at all)

- Remuneration and pay
- Job Security
- Working environment and conditions
- Working culture
- Work-life balance
- Ability to choose a work location
- Personal reasons (Family, partner, spouse, etc)
- Career pathway clarity

Planning to leave Medicine Permanently

You have indicated that you would like to leave medicine permanently. Which career field do you plan to join?

You have indicated that you would like to leave medicine permanently. Do you think that your degree is useful outside of medicine?

- Yes
- No

You have indicated that you would like to leave medicine permanently. How serious are you regarding this decision?

- It is just a thought in my mind
- I am actively searching for important and relevant info
- I am in the process of detailed planning
- I have made concrete steps or actions that will pave the way for this decision in the future

You have indicated that you would like to leave medicine permanently. Did you enter the medicine course voluntarily?

- Yes. But I did not understand the nature of the course or the profession
- Yes. But I was not aware of the career progression of this profession
- Yes. But I find the profession unsuitable for me
- Yes. I was unsure of what course to choose and I got an offer for medicine

- Yes. Other reasons
- No. I was pressured by my family
- No. I was pressured by social norms
- No. I did not get an offer for my desired course or university
- No. Other reasons

Reasons for leaving medicine permanently
Please indicate the level of importance of these factors in contributing to your decision (Very important, Important, Neutral, Not Important, Not important at all)

- Remuneration and pay at Houseman (HO) and Medical Officer (MO) level
- Remuneration and pay at Specialist and Consultant level
- Job Security
- Working environment and conditions
- Working culture
- Work-life balance
- Burnout
- Personal reasons (Family, partner, spouse, etc)
- Career pathway clarity
- High competition for career progression
- Ability to choose a work location

Do you think you will rejoin the healthcare sector in the future?

- Yes, but not as a doctor
- Yes, as a doctor
- Maybe
- No

<u>**Planning to leave Clinical Practice Temporarily (no more than 3 years)**</u>
You have indicated that you would like to leave medicine temporarily. How serious are you regarding this decision?

- It is just a thought in my mind
- I am actively searching for important and relevant info
- I am in the process of detailed planning
- I have made concrete steps or actions that will pave the way for this decision in the future

Reasons for leaving medicine temporarily (no more than 3 years)
Please indicate the level of importance of these factors in contributing to your decision (Very important, Important, Neutral, Not Important, Not important at all)

- To further studies (Masters, PhD, Certified courses, Short term programme)
- Research
- To build up a CV

- To try out other jobs or career
- To take a break and rest
- Burnout
- Health issues
- To work to save money
- To start a family
- Travel

Do you think you will rejoin the healthcare sector in the future?

- Yes, but not as a doctor
- Yes, as a doctor
- Maybe
- No

Section D: Specialisation

Do you plan to specialise in the future?
- Yes
- No

Are you certain about which specialty you wish to pursue?
- Very Certain
- Certain
- Neutral
- Uncertain
- Very Uncertain

Which specialty or specialties most interest you?
(You may choose up to a maximum of 3 specialties)

- Anaesthesiology
- Emergency Medicine
- Family Medicine
- Internal Medicine
- Nuclear Medicine
- Rehabilitation Medicine
- Sports Medicine
- Oncology
- Clinical Radiology
- Paediatrics
- General Pathology
- Anatomical Pathology
- Chemical Pathology
- Genetic Pathology
- Haematology
- Medical Microbiology
- Forensic Pathology
- Transfusion Medicine
- Psychiatry
- Public Health
- Obstetrics and Gynaecology
- Surgery
- Cardiothoracic Surgery
- Neurosurgery
- Paediatric Surgery
- Plastic Surgery
- Ophthalmology
- Otorhinolaryngology
- Orthopaedic Surgery
- Urology

Choice of Specialisation Pathway

- Postgraduate Programme (Masters) in local public universities
- Postgraduate Programme (Masters) in local private universities
- Skim Latihan Akademik Bumiputra (SLAB)/ Skim Latihan Akademik IPTA (SLAI)
- Parallel pathway
- Overseas Training

Reasons for deciding which specialty to pursue
Please indicate the level of importance of these factors in contributing to your decision (Very important, Important, Neutral, Not Important, Not important at all)

- Passion and Interest
- Remuneration and pay at the specialist and consultant level
- Job Demand
- Working environment and conditions
- Working culture
- Work-life balance
- Personal reasons (Family, partner, spouse, etc)
- Ability to choose a work location
- Career pathway clarity
- Opportunities to enter specialisation training programme (competition)
- Duration of specialisation training
- Quality of specialisation training
- Duration of service before entering a specialisation training programme
- Parallel pathway availability

Reasons for not wanting to specialise
Please indicate the level of importance of these factors in contributing to your decision (Very important, Important, Neutral, Not Important, Not important at all)

- Remuneration and pay during specialisation
- Stress during specialisation training
- Working environment and conditions
- Working culture
- Work-life balance
- To start a family
- Ability to choose a work location
- Career pathway clarity
- Opportunities to enter specialisation training programme (competition)
- Duration of Specialisation Training
- Quality of Specialisation Training

Certificate of Participation (Optional)

Name
Please provide your name as you wish to appear on the certificate of participation for this study. You may skip this section if you do not want to receive the certificate.

Email
The certificate of participation will be sent to this email. You may skip this section if you do not want to receive the certificate.
